# Early post-operative hemodynamic recovery in infants with congenital diaphragmatic hernia

**DOI:** 10.1007/s00431-026-06907-5

**Published:** 2026-04-20

**Authors:** Kamal Ali, Mahesh Nandunjappa, Abdulaziz Homedi, Fahad MS Arattu Thodika, Raghavendra Subba-Rao, Mohammed Almahdi, Saif Alsaif, Ibrahim Ali, Ravindra Bhat, Christopher Harris, Theodore Dassios, Anne Greenough

**Affiliations:** 1 Neonatal Intensive Care Department, King Abdulaziz Medical City, Riyadh, Saudi Arabia; 2 Neonatal Intensive Care Centre, King’s College Hospital, 4 Floor, Golden Jubilee Wing, Denmark Hill, London, SE5 9RS UK; 3 King Abdullah International Medical Research Centre, Riyadh, Saudi Arabia; 4 Department of Women and Children’s Health, School of Life Course Sciences, Faculty of Life Sciences and Medicine, King’s College London, London, UK; 5 College of Medicine, King Saud Bin Abdulaziz University for Health Sciences, Riyadh, Saudi Arabia

**Keywords:** Congenital diaphragmatic hernia, Pulmonary hypertension, Neonatal echocardiography, Ventricular dysfunction, Perioperative hemodynamics

## Abstract

Pulmonary hypertension and cardiac dysfunction contribute to morbidity in infants with congenital diaphragmatic hernia (CDH). Although surgical repair relieves thoracic compression, post-repair hemodynamic recovery remains incompletely understood. We aimed to characterize changes in ventricular function and pulmonary hypertension indices using paired echocardiography. This retrospective cohort study included 52 infants with CDH who underwent echocardiographic assessments 24 h before and 24–48 h after surgical repair. Paired analyses were performed for the continuous and categorical variables. Predefined abnormal thresholds were applied to assess post-repair normalization. Associations between changes in pulmonary hypertension markers and ventricular function were examined using correlation analysis. Significant improvements were observed in echocardiographic measures following repair. Ejection fraction increased from 62 to 72%, and tricuspid annular plane systolic excursion (TAPSE) increased from 7 to 12 mm (all *p* < 0.001). The right ventricular systolic pressure decreased from 30 to 24 mmHg, and the eccentricity index improved from 1.2 to 1.1 (both *p* < 0.001). Normal septal morphology was restored in 58.3% of infants, and right-to-left ductal shunting resolved in all affected cases. Reductions in the eccentricity index correlated with improvements in left ventricular output (*ρ* = − 0.31, *p* = 0.024), left ventricular longitudinal strain (*ρ* = 0.43, *p* = 0.005), TAPSE (*ρ* = 0.37, *p* = 0.006), and right ventricular output (*ρ* = − 0.37, *p* = 0.007).

*Conclusion*: Surgical repair of CDH is associated with early postoperative changes in echocardiographic indices of biventricular systolic performance and pulmonary vascular loading. Paired echocardiography allows quantification of early hemodynamic adaptation following repair.

**Table:** 

**What is Known:** • *Pulmonary hypertension and ventricular dysfunction are major contributors to early morbidity and mortality in infants with congenital diaphragmatic hernia (CDH).* •* The early hemodynamic impact of surgical repair, assessed using paired preoperative and postoperative echocardiography within individual infants, has not been well characterized.*	
**What is New:** • *Surgical repair of CDH is associated with early postoperative changes in echocardiographic indices of biventricular systolic performance, septal geometry, and pulmonary hypertension.* • *Changes in pulmonary vascular loading were closely associated with directional changes in ventricular systolic performance indices, highlighting the importance of ventricular-vascular interaction in early postoperative hemodynamic adaptation.*

**Supplementary Information:**

The online version contains supplementary material available at 10.1007/s00431-026-06907-5.

## Introduction

Congenital diaphragmatic hernia (CDH) is associated with major neonatal morbidity and mortality, driven primarily by pulmonary hypoplasia and abnormal pulmonary vascular development leading to marked pulmonary hypertension (PH) after birth [1, 2]. Persistent elevation of pulmonary vascular resistance increases the right ventricular (RV) afterload and contributes to hemodynamic instability during the early phase of postnatal transition, often necessitating vasoactive support, pulmonary vasodilator therapy, or extracorporeal life support [3]. Clinical cohort studies and registry data have demonstrated that the severity and early evolution of PH, rather than the diaphragmatic defect itself, are key determinants of the early clinical trajectory, need for extracorporeal membrane oxygenation (ECMO), and subsequent survival in infants with CDH [4–6].

Cardiac dysfunction is increasingly recognized as a central component of the pathophysiology of CDH rather than merely a secondary consequence of respiratory failure. Early postnatal impairment of RV and/or left ventricular (LV) performance is common and associated with greater disease severity and adverse outcomes [7, 8]. Elevated pulmonary vascular resistance increases RV afterload, promoting RV dilatation and systolic dysfunction, septal displacement, and impaired LV filling via ventricular interdependence, with consequent reduction in systemic output [9, 10]. In addition, LV hypoplasia and LV dysfunction related to altered fetal preload and reduced fetal left heart growth have been described in CDH, and can contribute to postnatal hemodynamic instability and pulmonary venous hypertension, with important implications for PH management [9, 11]. Collectively, these observations highlight the need for early targeted echocardiographic assessment of PH severity and biventricular performance in infants with CDH [10, 12].

Although surgical repair is a central management component in infants with CDH, its early hemodynamic impact has not been adequately described. In current practice, echocardiographic indicators of cardiopulmonary stability are increasingly being used to guide the timing of repair to minimize postoperative decompensation. However, detailed paired within-infant comparisons of ventricular function and pulmonary hypertension indices before and after surgical repair remain limited. As a result, the extent to which relief of pulmonary vascular load following repair is associated with the early recovery of ventricular performance has not been clearly established.

Therefore, this study aimed to characterize early hemodynamic changes following surgical repair of CDH using paired preoperative and postoperative echocardiography. Specifically, we assessed changes in right and left ventricular systolic function, interventricular septal configuration, pulmonary hypertension indices, and ductal shunt direction and examined the relationship between changes in pulmonary vascular load and recovery of ventricular function.

## Methods

### Study design and population

This retrospective observational cohort study was conducted at two tertiary neonatal surgical centers: King Abdulaziz Medical City, Riyadh, Saudi Arabia, and King’s College Hospital, London, UK. The study period was January 2016 to March 2025.

The study was registered with the Clinical Governance Department of King’s College Hospital, London. In accordance with the UK National Health Service Health Research Authority decision tool, the project was classified as a service evaluation and did not require review or approval by the Research Ethics Committee. For the Saudi Arabian center, ethical approval for retrospective data collection and analysis was obtained from the King Abdullah International Medical Research Centre (KAIMRC). Given the retrospective nature of this study and the use of fully de-identified clinical data, the requirement for informed consent was waived. All echocardiographic assessments were performed as part of routine perioperative clinical care, and no additional research-specific imaging was undertaken.

### Inclusion and exclusion criteria

Infants were eligible for inclusion if they had a confirmed diagnosis of CDH and underwent surgical repair during the initial neonatal admission. Perioperative echocardiography is part of routine clinical management in both centers for infants undergoing CDH repair. For the purposes of this analysis, infants were included if paired preoperative and postoperative echocardiographic studies were available within the predefined study window. To ensure temporal consistency, preoperative echocardiography was performed within 24 h prior to surgery and postoperative echocardiography within 24 to 48 h following repair.

Infants with major structural congenital heart disease were excluded, except for patent ductus arteriosus and patent foramen ovale. Echocardiographic studies were excluded from analysis if paired examinations were not available within the predefined perioperative window or if stored image-quality was inadequate for predefined measurements.

During the study period, 60 infants underwent CDH repair. All underwent echocardiographic assessment as part of routine perioperative care. Fifty-two had complete and analyzable paired studies within the predefined time window and were included in the final analysis. Eight were excluded due to incomplete paired timing or inadequate stored image quality.

### Echocardiographic assessment

Echocardiographic examinations were performed by neonatologists and pediatric cardiologists with formal training in targeted neonatal echocardiography and experience in neonatal functional echocardiographic assessment. Both centers operate established CDH programs with routine perioperative echocardiographic surveillance, ensuring standardized imaging protocols and expertise in the evaluation of pulmonary hypertension and ventricular function. Image acquisition followed standardized local protocols, informed by international recommendations for targeted neonatal echocardiography [13].

Left ventricular systolic function was assessed using ejection fraction, fractional shortening, and left ventricular output, indexed to body weight. The left ventricular longitudinal strain was assessed using speckle-tracking echocardiography when image quality permitted. Echocardiograms were acquired using GE ultrasound systems at King Abdulaziz Medical City and Philips ultrasound systems at King’s College Hospital. Strain analysis was performed offline from stored apical four-chamber loops using vendor-specific speckle-tracking software within each center and was undertaken only when digital loops were of adequate quality for endocardial tracking. Due to image quality limitations, strain measurements were available in 41 of 52 infants. Right ventricular systolic function was evaluated using tricuspid annular plane systolic excursion, right ventricular fractional area change, and right ventricular output indexed to body weight.

Pulmonary hypertension was evaluated by estimating the right ventricular systolic pressure from the tricuspid regurgitation jet, calculation of the eccentricity index at end systole, and qualitative assessment of interventricular septal configuration, categorized as normal, flat, or bowing. The ductal shunt direction across the patent ductus arteriosus was classified as right-to-left, bidirectional, or without a right-to-left component.

For paired analyses, normalization was defined as the transition from an abnormal value in the preoperative study to a value within the normal range in the postoperative study. For categorical variables, normalization was defined as the resolution of the abnormal category on the postoperative assessment.

For categorical analyses, predefined clinically established echocardiographic thresholds were applied as follows. Left ventricular systolic dysfunction was defined as ejection fraction (EF) < 55%, consistent with established pediatric echocardiographic conventions and American Society of Echocardiography (ASE) pediatric quantification recommendations [14, 15]. Elevated pulmonary pressure was defined as estimated right ventricular systolic pressure (RVSP) > 35 mmHg when derived from tricuspid regurgitation velocity, reflecting a commonly applied echocardiographic threshold for elevated pulmonary artery pressure in pediatric pulmonary hypertension literature and guideline frameworks [16, 17]. Septal flattening consistent with right ventricular pressure loading was defined using a systolic eccentricity index (EI) > 1.2. The EI was originally described as a quantitative marker distinguishing right ventricular pressure overload from volume overload [18] and has subsequently been applied in congenital diaphragmatic hernia (CDH) cohorts as a non-invasive marker of pulmonary vascular burden and outcome risk [19, 20]. Impaired left ventricular deformation was defined as global longitudinal strain (GLS) > − 18% (less negative), representing a conservative abnormality threshold anchored to pediatric normative data demonstrating mean GLS values approximating − 20% in healthy children [21]. Right ventricular systolic dysfunction was defined as tricuspid annular plane systolic excursion (TAPSE) < 8 mm. This threshold was selected in reference to published pediatric reference values demonstrating age-dependent normative TAPSE ranges and relatively low absolute values in neonates and young infants [22].

### Image analysis and data collection

All echocardiographic studies were analyzed offline using stored digital cine loops by two independent neonatologists with expertise in functional echocardiography. The raters were blinded to the infants’ clinical course. Only anonymized echocardiographic images and study identifiers were available during analysis.

Clinical data were extracted separately from electronic medical records and included gestational age, birth weight, sex, and mode of respiratory support. In addition to echocardiographic parameters, markers of pulmonary hypertension severity were recorded, including use of inhaled nitric oxide (iNO), need for escalation to milrinone therapy, and requirement for extracorporeal membrane oxygenation (ECMO). Oxygenation index measurements were not systematically recorded at standardized time points relative to iNO initiation; therefore, formal classification of refractory PPHN based on OI response criteria could not be uniformly applied. Data extraction was performed independently by two investigators and cross-checked to ensure completeness and consistency across both study centers.

### Statistical analysis

All statistical analyses were performed using the IBM SPSS Statistics version 23 (IBM Corp., Armonk, NY, USA). Preoperative and postoperative echocardiographic measurements were treated as paired observations for each infant. Normality of paired differences for continuous variables was assessed using the Shapiro–Wilk test. As paired differences did not meet the assumptions of normality, continuous variables were presented as medians with interquartile ranges, and comparisons between preoperative and postoperative values were performed using the Wilcoxon signed-rank test.

Paired categorical variables were analyzed using methods appropriate for dependent data. Binary outcomes, including right-to-left ductal shunting and abnormal versus normal echocardiographic classifications, were analyzed using the McNemar’s test. Ordered categorical variables with more than two levels, including interventricular septal morphology (normal, flat, and bowing), were analyzed using the marginal homogeneity test. For analyses of postoperative normalization, McNemar’s test was used to evaluate paired changes in abnormal status from preoperative to postoperative assessment.

Predefined clinical thresholds were applied to classify echocardiographic parameters as normal or abnormal, including ejection fraction < 55%, tricuspid annular plane systolic excursion < 8 mm, eccentricity index > 1.2, and right ventricular systolic pressure > 35 mm Hg. For each threshold, the proportion of infants with abnormal values before and after surgery was calculated, and the proportion demonstrating postoperative normalization was determined. Changes in paired proportions were evaluated using McNemar’s test.

To examine the associations between changes in pulmonary vascular load and recovery of ventricular function following surgical repair, change scores for right ventricular systolic pressure and eccentricity index were correlated with changes in ventricular systolic indices using Spearman rank correlation coefficients.

To evaluate whether defect laterality influenced the magnitude of perioperative hemodynamic change, multivariable linear regression models were constructed using change scores (postoperative minus preoperative values) for each echocardiographic parameter as dependent variables. Hernia laterality (right- vs left-sided) was included as the primary predictor, and models were adjusted for gestational age and the baseline (preoperative) value of the same echocardiographic parameter. Regression estimates are reported as *β* coefficients with 95% confidence intervals. Statistical significance was defined as a two-sided *p*-value < 0.05. Results of these models are presented in Supplementary Table S1.

## Results

Fifty-two infants with CDH underwent paired preoperative and postoperative echocardiographic assessments (Table 1). Infants were predominantly born at term, with a median gestational age of 38 weeks and a median birth weight of 2510 g. Left-sided diaphragmatic defects predominated (76.9%) and liver herniation was present in nearly half of the cohort. Surgical repair was performed at a median postnatal age of 7 days. Preoperatively, 55.8% of infants required high-frequency oscillatory ventilation. Markers of pulmonary hypertension and cardiovascular instability were common, with 65.4% receiving inhaled nitric oxide and 78.8% requiring preoperative inotropic or vasoactive support.

**Table 1:** Baseline clinical characteristics of infants with CDH. Data are demonstrated as median (IQR) or *n* (%)

Gestational age (weeks)	38 (35, 39)
Birth weight (grams)	2510 (2130, 3000)
Male sex	22 (42.3%)
Left-sided CDH	40 (76.9%)
Right-sided CDH	12 (23.1%)
Liver herniation	25 (48.1%)
Age at surgical repair (days)	7 (4–10)
Conventional mechanical ventilation	23 (44.2%)
High-frequency oscillatory ventilation	29 (55.8%)
Use of inhaled nitric oxide preoperatively	34 (65.4%)
Use of inotropic or vasoactive support preoperatively	41 (78.8%)
Study center	
King Abdulaziz Medical City, Riyadh	36 (69.2%)
King’s College Hospital, London	16 (30.8%)

Surgical repair was associated with significant improvements in all continuous measures of biventricular systolic function and pulmonary hemodynamics (Table 2). The left ventricular systolic performance improved with increases in ejection fraction, longitudinal strain, and ventricular output (all *p* < 0.001). The right ventricular systolic indices demonstrated parallel recovery, including increases in tricuspid annular plane systolic excursion, fractional area change, and right ventricular output, along with a reduction in the eccentricity index and right ventricular systolic pressure (all *p* < 0.001). Categorical echocardiographic measures showed concordant postoperative improvements. The normal septal morphology increased from 30.8% preoperatively to 71.2% after repair, with complete resolution of septal bowing (*p* < 0.001). Right-to-left ductal shunting resolved in all affected infants following surgery (*p* < 0.001).

**Table 2:** Paired preoperative and postoperative echocardiographic parameters in infants with CDH

Parameter		Preoperative median (IQR)	Postoperative median (IQR)	*p*-value
Continuous echocardiographic measures				
Ejection Fraction (%)		62 (57–66)	72 (66–78)	<0.001
LV Longitudinal Strain (%)		–18.0 (–19.0 to –17.0)	–20.0 (–21.0 to –19.0)	<0.001
LV Output (mL/kg/min)		200 (185–240)	230 (200–260)	<0.001
Fractional Area Change (%)		44 (40–46)	46 (44–49)	<0.001
Fractional Shortening (%)		33 (28–36)	39 (36–44)	<0.001
TAPSE (mm)		7 (6–7)	12 (10–12)	<0.001
RV Output (mL/kg/min)		250 (220–300)	280 (240–310)	<0.001
EI		1.2 (1.1–1.3)	1.1 (1.0–1.1)	<0.001
RV Systolic Pressure (mmHg)		30 (24–45)	24 (15–25)	<0.001
Variable	Category	Preoperative *n* (%)	Postoperative n (%)	*p*-value
Categorical echocardiographic measures				
Septal Morphology(1 = normal, 2 = flat, 3 = bowing)	Normal	16 (30.8%)	37 (71.2%)	<0.001
	Flat	32 (61.5%)	15 (28.8%)	
	Bowing	4 (7.7%)	0 (0%)	
PDA Shunt Direction(1 = right-to-left, 2 = no right-to-left)	Right-to-left	18 (34.6%)	0 (0%)	<0.001
	No right-to-left		52 (100%)	

*EI* eccentricity index, *TAPSE* tricuspid annular plane systolic excursion, *RV* right ventricular, *LV* left ventricular, *PDA* patent ductus arteriosus

A greater reduction in right ventricular systolic pressure was associated with an increase in left ventricular output (*ρ* = − 0.43, *p* = 0.001) and improvement in the eccentricity index (*ρ* = 0.31, *p* = 0.024) (Table 3). Reductions in the eccentricity index correlated with improvements in left ventricular longitudinal strain (*ρ* = 0.43, *p* = 0.005), tricuspid annular plane systolic excursion (*ρ* = 0.37, *p* = 0.006), and right ventricular output (*ρ* = − 0.37, *p* = 0.007). No significant associations were observed between changes in pulmonary hypertension indices and conventional systolic measures including ejection fraction, fractional area change, or fractional shortening.

**Table 3:** Correlations between changes in pulmonary hypertension indices and ventricular function following CDH repair

Change in ventricular function	ΔRVSP *ρ*	*p* value	ΔEI *ρ*	*p* value
Δ Left ventricular output (LVO)	− 0.43	0.001	− 0.31	0.024
Δ Left ventricular longitudinal strain	+ 0.17	0.274	+ 0.43	0.005
Δ Ejection fraction (EF)	− 0.20	0.149	− 0.07	0.616
Δ Fractional shortening (FS)	− 0.05	0.710	+ 0.02	0.912
Δ Fractional area change (FAC)	+ 0.17	0.216	+ 0.22	0.116
Δ Tricuspid annular plane systolic excursion (TAPSE)	− 0.17	0.215	+ 0.37	0.006
Δ Right ventricular output (RVO)	+ 0.08	0.559	− 0.37	0.007
Δ Eccentricity index (EI)	+ 0.31	0.024	—	—

Spearman rank correlation coefficients (*ρ*) are shown. *Δ* indicates postoperative minus preoperative change. Correlation between change in eccentricity index and itself is not shown

Significant postoperative improvement was observed (Table 4). Normalization occurred in 77.8% of the infants with reduced ejection fraction (7 of 9; *p* = 0.016) and in 97.1% of those with reduced tricuspid annular plane systolic excursion (34 of 35; *p* < 0.001). Elevated right ventricular systolic pressure normalized in 75% of the affected infants (15 of 20; *p* < 0.001), while the abnormal eccentricity index resolved in 87.0% (20 of 23; *p* < 0.001). Abnormal septal morphology normalized in 58.3% of the infants (21 of 36; *p* < 0.001). All infants with right-to-left ductal shunting demonstrated complete postoperative resolution (*p* < 0.001). Although the left ventricular longitudinal strain improved in over half of the affected infants (55.6%), this did not reach statistical significance (*p* = 0.063).

**Table 4:** Normalization of echocardiographic abnormalities following surgical repair of congenital diaphragmatic hernia

Parameter (abnormal threshold)	Preoperative abnormal *n* (%)	Postoperative abnormal *n* (%)	Normalized *n*/*N* (%)	McNemar *p* value
Ejection fraction < 55%	9 (17.3)	2 (3.8)	7/9 (77.8)	0.016
LV longitudinal strain > − 18%*	9/41 (22.0)	4/41 (9.8)	5/9 (55.6)	0.063
TAPSE < 8 mm	35 (67.3)	1 (1.9)	34/35 (97.1)	< 0.001
RV systolic pressure > 35 mmHg	20 (38.5)	5 (9.6)	15/20 (75.0)	< 0.001
Eccentricity index > 1.2	23 (44.2)	3 (5.8)	20/23 (87.0)	< 0.001
Abnormal septal morphology†	36 (69.2)	15 (28.8)	21/36 (58.3)	< 0.001
Right-to-left PDA shunt	18 (34.6)	0 (0)	18/18 (100)	< 0.001

^*^Left ventricular longitudinal strain was available in 41 infants due to limitations in speckle tracking image quality; more negative values indicate improved systolic function

^†^Abnormal septal morphology includes flat or bowing interventricular septal configuration

Markers of pulmonary hypertension severity decreased significantly following surgical repair (Table 5). The proportion of infants receiving inhaled nitric oxide declined from 80.8% preoperatively to 13.5% postoperatively (*p* < 0.001). Milrinone use decreased from 23.1 to 9.6% (*p* = 0.016), and the requirement for other vasoactive or inotropic support decreased from 76.9 to 11.5% (*p* < 0.001). No infant required extracorporeal membrane oxygenation at any stage of management.

**Table 5:** Pulmonary hypertension severity markers before and after CDH repair

Variable	Preoperative *n* (%)	Postoperative *n* (%)	*p*-value*
Inhaled nitric oxide (iNO)	42 (80.8%)	7 (13.5%)	< 0.001
Milrinone therapy	12 (23.1%)	5 (9.6%)	0.016
Other inotropic/vasoactive support*	40 (76.9%)	6 (11.5%)	< 0.001

^*^Includes vasoactive agents such as dopamine, dobutamine, epinephrine, or norepinephrine

Hernia laterality was not independently associated with the magnitude of perioperative hemodynamic change after adjustment for gestational age and baseline values (Supplementary Table S1). There was no significant association between right-sided hernia and change in right ventricular systolic pressure, tricuspid annular plane systolic excursion, left ventricular output, or ejection fraction. A small association was observed between right-sided hernia and change in eccentricity index (*β* = − 0.06, 95% CI − 0.12 to − 0.00; *p* = 0.041).

## Discussion

In this retrospective observational cohort study, we assessed early hemodynamic changes following CDH repair using paired preoperative and postoperative echocardiographic measurements. We observed consistent early postoperative changes across multiple echocardiographic domains, including indices of biventricular systolic performance and pulmonary vascular loading. Across continuous echocardiographic measures, statistically significant directional changes were observed in left ventricular systolic indices and right ventricular longitudinal systolic excursion, accompanied by reductions in surrogate markers of pulmonary vascular load. Although median ejection fraction values were within the conventional normal range at both time points, a subset of infants had reduced preoperative systolic function that normalized following repair, suggesting resolution of loading-related impairment rather than development of supranormal function. This pattern is consistent with the recognized pathophysiology of CDH, in which elevated pulmonary vascular resistance increases right ventricular afterload, promotes interventricular interaction, and impairs systemic output. Surgical repair and subsequent stabilization appear to be associated with partial normalization of these loading conditions, which may explain the observed shifts in systolic performance indices [23, 24]. These findings likely reflect alterations in right ventricular loading conditions rather than intrinsic enhancement of myocardial contractility.

The correlation analyses in our study further supported the notion that ventricular interdependence is an important mechanism underlying postoperative hemodynamic improvement. Reductions in estimated right ventricular systolic pressure were associated with improvements in systemic forward flow and normalization of septal configuration, whereas decreases in the eccentricity index correlated with broader recovery across deformation and right ventricular systolic parameters. These findings are in agreement with the existing CDH literature, which emphasizes that indices of ventricular-vascular interaction and echocardiographic indices that reflect ventricular loading and septal mechanics have prognostic value in the population of infants with CDH [25, 26].

When we applied predefined abnormal thresholds in our cohort, we observed high rates of postoperative normalization across the key echocardiographic markers of pulmonary hypertension and ventricular dysfunction, including complete resolution of right-to-left ductal shunting in infants in whom it was present preoperatively. Although median strain values were within accepted normal ranges at both time points, a subset of infants demonstrated preoperative strain impairment that resolved following repair. These threshold-based findings are consistent with prior work demonstrating that both the persistence and severity of pulmonary hypertension on echocardiography are strongly associated with short-term outcomes in CDH, including survival and postoperative course [5, 27]. Similar observations regarding the prognostic importance of pulmonary hypertension severity and response to inhaled nitric oxide have been reported in infants with CDH [28].

In multivariate linear regression models examining postoperative-minus-preoperative change, hernia laterality was not associated with improvement in RVSP, TAPSE, LVO, or EF after adjustment for gestational age and baseline values. Right-sided defects were associated with a small difference in EI change, with less reduction in EI compared with left-sided defects, although the magnitude of this association was modest. Overall, these analyses suggest that the pattern of perioperative hemodynamic change observed in this cohort was broadly similar across defect laterality.

Formal classification of refractory persistent pulmonary hypertension of the newborn (PPHN) using oxygenation index response criteria could not be applied due to the retrospective design and variability in arterial blood gas timing relative to inhaled nitric oxide initiation. Classical definitions of refractory PPHN rely on failure of oxygenation index improvement after inhaled nitric oxide therapy in prospective neonatal pulmonary hypertension studies [29, 30]. In this cohort, a high proportion of infants required preoperative inhaled nitric oxide and milrinone, indicating a significant pulmonary hypertension burden. The importance of targeted pulmonary vasodilator therapy in infants with CDH-associated pulmonary hypertension has also been highlighted in recent clinical reports evaluating pharmacologic management strategies [31]. Following surgical repair, the requirement for pulmonary vasodilator and vasoactive support decreased significantly, in parallel with improvements in echocardiographic markers of pulmonary pressure and ventricular interaction. Previous CDH studies have shown that persistent pulmonary hypertension and ongoing need for vasodilator therapy are associated with adverse short-term outcomes [12]. The observed reduction in pharmacologic support therefore provides clinical context for the echocardiographic changes. No infant required extracorporeal membrane oxygenation support.

Previous studies have reported that echocardiography can identify high-risk CDH infants and predict outcomes using markers of pulmonary hypertension and ventricular dysfunction, with early studies focusing on pulmonary pressure estimates and later studies incorporating measures of ventricular performance and ventricular-vascular interaction [27, 32]. Our paired preoperative and postoperative measurements enrich this literature by focusing on within-infant changes across a predefined perioperative interval. This approach demonstrates that several clinically meaningful echocardiographic abnormalities can improve over a relatively short time frame following surgical repair in appropriately selected infants rather than representing fixed markers of disease severity.

Taken together, these findings suggest that serial echocardiographic assessment may help characterize early perioperative physiological adaptation in infants with CDH. The observed early changes in pulmonary hypertension indices and ventricular performance suggest that targeted echocardiography may contribute to hemodynamic assessment and decision-making during the perioperative period. Incorporating standardized echocardiographic indices into routine perioperative assessment may help characterize early physiological trajectories and guide closer cardiopulmonary monitoring where appropriate.

The strengths and limitations of our study merit consideration. Key strengths include the paired preoperative and postoperative echocardiographic design, which enabled within-infant assessment of hemodynamic change across a defined perioperative interval, and the use of standardized perioperative imaging time points. The within-infant comparison minimized confounding related to inter-individual differences in gestational age, defect characteristics, and baseline cardiopulmonary status. The study incorporated multiple echocardiographic measures of ventricular systolic function, septal geometry, and pulmonary hypertension, providing a structured evaluation of cardiopulmonary physiology in infants managed at two specialized tertiary CDH centers.

Several limitations should also be acknowledged. Doppler-derived cardiac output measurements are inherently load-dependent and subject to technical variability; however, the paired within-infant design reduces the impact of systematic measurement bias when comparing preoperative and postoperative values within the same infant. The retrospective design introduces potential selection bias toward infants with available high-quality paired echocardiographic examinations. In addition, speckle-tracking–derived strain measurements were not available in all infants due to image-quality limitations, reducing the sample size for deformation analyses. Comprehensive assessment of left ventricular diastolic function was not systematically available and represents an important area for future prospective study.

Although measurements were performed by experienced operators using standardized acquisition protocols and blinded offline analysis with consensus review, formal inter-observer variability statistics (e.g., intraclass correlation coefficients) were not calculated. Furthermore, statistical significance should not be interpreted as evidence of uniform clinical benefit at the individual patient level, as many observed changes reflect load-sensitive physiological shifts rather than intrinsic myocardial recovery. Finally, the analysis focused on early postoperative changes and was not designed to determine whether these echocardiographic findings translate into differences in long-term respiratory morbidity or survival outcomes. Given the retrospective design and modest sample size, these findings should be interpreted with caution and considered hypothesis generating, warranting confirmation in larger prospective multicenter studies.

## Conclusion

In infants with congenital diaphragmatic hernia, surgical repair was associated with early improvement in biventricular systolic function, septal geometry, and echocardiographic markers of pulmonary hypertension as demonstrated by paired preoperative and postoperative assessments. These changes are consistent with relief of right ventricular pressure loading and improved ventricular interaction during the early postoperative period. Paired echocardiographic evaluation provides a structured approach to quantify early hemodynamic recovery after CDH repair. Given the retrospective design and modest sample size, these findings should be interpreted as exploratory and hypothesis generating and warrant confirmation in larger prospective studies.

## Supplementary Information

Below is the link to the electronic supplementary material.

ESM 1 Supplementary Material 1 (DOCX 14.4 KB)

## Data Availability

The datasets generated and analyzed during the current study are available from the corresponding author upon reasonable request, subject to appropriate institutional approvals.
