# Supplementary material for: Early post-operative hemodynamic recovery in infants with congenital diaphragmatic hernia
Source: Eur J Pediatr. 2026 Apr 20;185(5):281. doi: 10.1007/s00431-026-06907-5 (PMC13095933; doi:10.1007/s00431-026-06907-5)
Supplement: Supplementary file 1 — Supplementary Material 1 (DOCX 14.4 KB) [file 431_2026_6907_MOESM1_ESM.docx]

# **Supplementary Table S1. Multivariable linear regression: association between hernia laterality and hemodynamic improvement**

| Outcome (post–pre) | β for right-sided hernia (95% CI) | p-value |
| --- | --- | --- |
| ΔRVSP (mmHg) | -0.14 (-4.97 to 4.69) | 0.954 |
| Δ Eccentricity index (EI) | -0.06 (-0.12 to -0.00) | 0.041 |
| Δ TAPSE (mm) | -0.14 (-1.20 to 0.93) | 0.799 |
| Δ Left ventricular output (mL/kg/min) | 10.25 (-20.32 to 40.82) | 0.503 |
| Δ Ejection fraction (%) | 3.60 (-2.33 to 9.52) | 0.228 |
